# Sexual Orientation and Gender Identity Data in Oncology: Thematic Analysis From a National Qualitative Interview Study

**DOI:** 10.1002/cam4.71360

**Published:** 2025-11-20

**Authors:** Beck Gold, Dana Rosenberg, Megan A. Mullins, Milena E. Insalaco, Edward J. Miech, Charles Kamen, Mandi L. Pratt‐Chapman

**Affiliations:** ^1^ Department of Medicine GW Cancer Center, the George Washington University (GWU) School of Medicine and Health Sciences Washington DC USA; ^2^ O'Donnell School of Public Health, Simmons Comprehensive Cancer Center University of Texas (UT) Southwestern Medical Center Dallas Texas USA; ^3^ University of Rochester (UR) Medical Center Rochester New York USA; ^4^ Indiana University School of Medicine Department of Emergency Medicine Indianapolis Indiana USA

**Keywords:** gender identity, implementation, qualitative, sexual orientation

## Abstract

**Importance:**

Sexual orientation and gender identity (SOGI) data are vital, but inconsistently collected among cancer centers.

**Objective:**

This study aims to identify themes from a large set of interviews regarding the collection of sexual orientation and gender identity (SOGI) data in oncology practice.

**Setting:**

Oncology care settings in diverse geopolitical regions in the United States.

**Participants:**

Sixty‐two semi‐structured interviews across 23 cancer centers in the United States were conducted from September 1, 2022 to August 31, 2023. Interview transcripts were transcribed and double‐coded using thematic analysis and emergent coding.

**Main Outcomes and Measures:**

We used inductive, open coding thematic qualitative analysis with no predetermined constructs.

**Results:**

Key themes included: (1) barriers to SOGI data collection include stigma and bias, generational and geopolitical resistance, fragmented workflows, competing priorities and institutional inertia; (2) the importance of staff exposure to lesbian, gay, bisexual, queer, and intersex (LGBTQI) individuals in understanding SOGI's clinical relevance; and (3) structural challenges to equitable care for transgender patients, including electronic health record (EHR) limitations and billing issues. Taken together, these findings reveal a complex interplay of sociocultural, institutional, and systemic barriers that impede routine SOGI data collection in cancer care.

## 1 Introduction

Over the last decade, there have been numerous calls to collect sexual orientation and gender identity (SOGI) data in clinical practice to assess and address health disparities among lesbian, gay, bisexual, transgender, queer, and intersex (LGBTQI) people [1, 2, 3]. Specific to oncology, the American Society of Clinical Oncology (ASCO) has recommended SOGI data collection along with enhanced patient education and support, workforce diversity representation, quality improvements, and research investments to improve care and outcomes for LGBTQI people [4].

Yet SOGI data collection is not routine in oncology practice [5]. Studies show that both sex and gender are vital and distinct components of patient information that influence medical decision‐making in healthcare practice [6, 7, 8, 9]. For example, common musculoskeletal pathologies disproportionately affect people assigned female sex at birth, including osteoporosis‐related pathologic fractures [10]. Gender‐ and sex‐specific care is ultimately important in informing evidence‐based practices, identifying morbidity and mortality trends, and for providing more personalized and inclusive treatment. In the context of cancer care, there is evidence of sex‐based differences in nonreproductive cancers [11]. This highlights the need for stratification of sex, gender, and sexual orientation in current research to inform clinical guidelines.

In prior research, we found that practices at the forefront of SOGI data collection in oncology care settings tend to have strong leadership support, committed resources, and clinicians who value SOGI data as relevant to cancer supportive care and medical management considerations [8, 12]. In a separate deductive qualitative analysis, we found that systematic collection of SOGI data in oncology practices was associated with state or institutional mandates [13, 14], forced workflows, and redundant data collection processes [14]. The present study was conducted to explore emergent themes that fell outside the framework of our prior deductive analysis, drawing from the largest known qualitative dataset examining SOGI clinical data collection across the United States [8].

## 2 Methods

### 2.1 Study design

This is a qualitative, multi‐site interview study using emergent thematic analysis.

### 2.2 Ethical Review

This study was approved by the GW Institutional Review Board (NCR224177), UT Southwestern Institutional Review Board (STU‐2022‐1122), and UR Research Subjects Review Board (STUDY00007236). Participants were asked to complete a screening survey in REDCap that indicated that completion of demographic information served as consent for the study. Verbal consent to proceed with and record each interview was obtained prior to conducting the interviews.

### 2.3 Study Population

From September 2022 to August 2023, PhD‐prepared qualitative researchers conducted interviews (*n* = 62) from purposively sampled oncology practices (*n* = 23) across the United States. These institutions were diverse in geographic location, urbanicity, and academic setting. We ceased recruiting sites when data saturation was reached. Given there were no predetermined outcomes for this study, data saturation was defined as reaching a point at which no new themes were identified from additional interviews. We attempted to interview three interest holders (clinician, staff, administrator) per setting to obtain different perspectives on SOGI data (range *n* = 1–4 interviews per setting).

### 2.4 Recruitment

An email was sent to ASCO members (i.e., oncologists) requesting participants to complete a REDCap screening questionnaire that queried respondents on geographic location and practice setting (private vs. academic). Nine oncologists were recruited for interviews. Oncologists were asked to identify an administrator and a staff member who would also be willing to be interviewed at their site. Eight additional institutions were added from a separate study that asked similar questions (NCORP grant UG1CA189867). ASCO membership lists were reviewed to recruit five additional centers to maximize the diversity of geographic representation and SOGI data collection practices based on comparison to already recruited sites.

### 2.5 Data Collection

A semi‐structured interview guide [14] was used to explore current SOGI data collection processes and contextual factors relevant to data collection. The guide was iteratively modified during the period in which interviews were conducted to ensure data saturation. Interviews were approximately 60 min in duration. Zoom recordings of interviews were stored on the GW Box system. Participants were emailed a summary of major findings from their respective interviews in order to confirm accuracy.

### 2.6 Data Analysis

Interviews were transcribed verbatim via Zoom and manually checked for accuracy. Elsewhere, we have published deductive qualitative findings of *facilitators* for SOGI data collection based on major domains of the Consolidated Framework for Implementation Research (CFIR) [14]. Constant comparative analysis was used to assess data saturation based on the CFIR. In the present study, we report on inductive, open‐text coding of *barriers* to SOGI data collection. Using thematic analysis, two research team members identified emergent themes using NVivo and Dedoose software. Consensus meetings were held to resolve conflicting codes. When needed, the senior author resolved disputes. Data saturation was determined when no new themes arose in the interview transcripts.

### 2.7 Data Reporting

We referenced the Consolidated Criteria for Reporting Qualitative Research checklist to ensure our published results adhered to reporting guidelines.

### 2.8 Site Characteristics

We purposively selected cancer centers that varied based on size and nature of center, geography, and self‐reported collection of SOGI. Institutional characteristics are reported based on the consensus of at least two respondents from that site or by combining partial data from different respondents from the same site. Of the 23 participating sites, seven sites systematically collected, 11 sites partially collected, and five sites did not collect SOGI data. Regionally, sites were from the North (*n* = 6), South (*n* = 5), Midwest (*n* = 7), and West (*n* = 5). Eighteen sites were urban, four were suburban, and one was rural. Fourteen centers were academic cancer centers. Among the 62 interviewees, there was a mix of straight (*n* = 36), LGBTQI (*n* = 9), and undisclosed (*n* = 17) sexual orientations (Refer to deductive analysis for full demographic data) [14].

### 2.9 Researcher Reflexivity

The authors include four PhD‐trained researchers in the areas of translational research, psychology, epidemiology, and education. One author holds a nursing license and a master's in public health. Two authors are medical and public health students, respectively. Five of the seven authors identify within the LGBTQI+ community, which provided complementary emic and etic approaches to the research.

## 3 Results

Three major themes emerged from our analysis: (1) barriers to SOGI data collection, (2) importance of staff exposure in understanding the clinical relevance of SOGI, and (3) barriers to quality care for transgender patients. Themes and subthemes are described below with supporting quotations; see Table 1 [hereafter, only quotation (Q) number will be referenced].

**TABLE 1:** Representative quotations illustrating themes related to barriers in sogi data collection.

Theme/Subtheme	Quote ID	Type of cancer center	Size of center	Quotation
Barriers to SOGI data collection				
Stigma and bias	1	Academic	Medium	To be transparent, I think that… some of the staff would probably be uncomfortable and unfamiliar with this and that implicit bias kind of comes in, you know, and maybe even not even implicit. I mean, there could be bias, among particular groups of people. Right. Um, that might look at things differently than, you know, maybe me or you…or whoever. So I think that's one, one hurdle is, is… the bias, the comfortability is the second.
	2	Academic	Large	I think …patients may not be totally forthcoming with that information if they feel like they might be judged in some way or might or it could be used against them … in terms of… the way they're treated by certain members of the team… I think that's the, the social stigma around it on both sides … could be the… biggest barrier.
Generational variations in comfort discussing SOGI	3	Academic	Medium	We have an older patient population, and I think sometimes they're, they're confused by some of the questions that we ask.
	4	Academic	Large	Not to be ageist, but I think there definitely a, is a group of kind of older doctors that it's the same way. Their view of gender and sexual practice and stuff is just so narrow. They have a harder time talking about those things. They're great talking about it within the scope of what they understand, but I think their scope is narrow.
	5	Academic	Medium	It seems to be my, my younger generation that's saying, Hey, you know what, this is how I identify and I just want people to know that.
	6	Nonacademic	Small	My scribes are all Gen Z, they're, they're like <laugh>, they were so…like 70% of them are bisexual, you know, but it's not even a thing anymore.
	7	Academic	Large	I think with anything you, you're gonna have, you know, um, you know, resistance. But I think it's very, very minimal, you know, if any at all.
Geopolitical variations in comfort discussing SOGI	8	Nonacademic	Small	The community still very much has a heteronormative, Judeo‐Christian kind of approach. You know, southern white … I think that if you individually polled any of my partners, they would say they would kinda give the lip service. Of course, we're open accepting to everyone, but they're still joking in the background about, you know, patients that claim they're, claim they're trans or kind of dead naming or … misgendering, intentional misgendering.
	9	Academic	Large	I think part of it is geographically, so I'm, I'm in Midwest now. I was in [southern state] before this [southern state] was worse. Um, but at the same time I interviewed for jobs on the west coast and my interview in [city] was the only place they misgendered my partner the entire time. I was like, really? Really? We're in [city] and you're misgendering my partner? How hard is this?
Polarized Sociopolitical context	10	Academic	Large	You've got like on one end, this movement that's bringing people from the marginalized sectors to be more empowered to stand up for themselves. But then, you've got the other political powers, “Don't say gay,” right? Things like that. Yes, we're [northern state], but [northern state] can turn red at any minute. So, people are well aware of that. I think the political environment also may hinder people's willingness to go there, express that.
	11	Academic	Large	I think unfortunately it's now become this cultural value to discriminate against queer people in a way that is new for me. I think it's gonna get maybe a little bit worse before it gets better. But in terms of data collection, if it's on the form and it's part of your job, I think at least the data will be collected even if like the cultural shift is probably going to regress a little bit in the short term.
	12	Academic	Large	People got really, really concerned, because we tried to educate them… don't automatically call someone Mr. or Mrs. Ask them, um, what they, how they would like to be addressed. We had people who got really bent out of shape thinking, oh my gosh, I'm gonna ask my 95‐year‐old patient what his sexual orientation is and his gender identity is. And, and we were like, yeah you are… people had such a hard time buying into that.
	13	Nonacademic	Medium	We're so homogeneous, you know, which is not something we're proud of, but it's very interesting because there's a lot of confusion among patients, like, ‘why am I being asked that?’ So there's some, uh, still pushback among patients in our community.
	14	Academic	Large	We have a lot of elderly patients… they're not necessarily accustomed to this question, and that it could be very off‐putting to them… So I think there was just a lot of concern of what would the patient experience be like in being asked these questions.
Heterogeneous, fragmented data collection and access	15	Academic	Large	Some [Some SOGI data collection] is paper, some is the front desk staff, some is electronic… I wonder if it would be probably the easiest and you'd get the most correct answers if the patient is allowed to provide their own data, whether it's through an intake website or, electronically through an intake form rather than coming through a front desk staff who's asking questions… it's very disjointed right now.
	16	Academic	Large	It's such a kind of disjointed medical record system right now that it, there's not a lot of crosstalk between the two different parts.
	17	Academic	Large	I think Epic will help because there are fields for that information, So it gets carried forward. I think we're still struggling with the transition from paper into Epic. Who is gonna put all that data in unless you have like an electronic form? And if we transition to… an electronic form that automatically went into Epic or we had a system to get that into Epic, that would be better.
Competing priorities	18	Academic	Large	So, do I think we can make these changes as a policy change to our demographic collection, asking people about identity…it is difficult now because there are other priorities. Social needs, for instance, is a huge priority. Social determinants of health, getting housing, food, so I think that's why this area, it doesn't have as much prominence.
	19	Academic	Large	When you're going through the registration, … they need to meet time quotas. And once you have the standardized process of… you know, race, ethnicity, demographic information, now all of a sudden you have the gender identity, all this other information being collected… [You need an] …extension of the time.
Institutional inertia	20	Academic	Large	Institutional inertia and a bunch of attorneys. I'm pretty sure, um, it's usually those two things. I really did try and then they said that they were already working on an overhaul and then I asked if I could see it and they said no. And then I asked, can we just change ours? And they said no, cause legal has to look at it. It just, it became a whole [thing], because… they want the same form for the entire cancer center. It's just institutional inertia ‐ I think is most of it.
	21	Academic	Large	I remember we wanted to update our form recently to have it be more inclusive language and something as simple as updating a form‐you know, there, it was like a bunch of red tape. You had to get it approved, you had to get the language vetted, you had to get it approved by like multiple different committees. We had to justify what we were gonna do with this information. So something as simple as wanting to have a form be more inclusive took a little bit of time…if we were using Epic and it was all standard from the top, it would've been much easier.
Importance of exposure to understanding SOGI relevance				
Varying perceptions of SOGI relevance to clinical practice	22	Academic	Medium	It really does not matter to me, they can be two purple unicorns… I honestly think [level of receptivity is only 40%], because it really doesn't matter to what we do, it matters to the patient as a whole, of course, but it really does not matter for what we do, and I think that those people that maybe would not be interested in collecting the data simply are less interested in the social aspect of the patient's life. … Which you know some people are, I just really like to know, like how many toes your cat has.
	23	Academic	Large	It's not obviously the most critical thing for me to [be able to] deliver care to that patient at that moment, so maybe it's not the most critical thing. And, in the hustle and bustle, maybe it gets left on the side.
Need for education on SOGI data collection	24	Academic	Large	What I've seen is that when people are questioning this, you know, why we're doing this, it comes from a place of them not having exposure to this before in the background, and when you know, I go and I provide the information based on what I've been trained on… I feel like it's an easier way to get to them. And, I mean they could still be skeptical… But I think when we say that you know at the end of the day, this may help our patients, and that's you know what we're in the business of doing.
	25	Academic	Large	Providers were not comfortable around asking people's sexual identity…[I]t was very surprising to us. And so we actually have done quite a few education sessions. We've brought in gay men and physicians… to have them speak to the nurses and social workers [and] even the physicians.
Emerging perception of the relevance of SOGI through exposure	26	Academic	Medium	No [no barriers at my practice to being able to collect SO], my only thought would be, is why we would ask, and in the way where … it really doesn't play a role in caring for our patient, because the only way it would really matter, and I thought about this today, is a transgender patient with breast cancer and the endocrine therapy, because we use different endocrine therapy for biologic males then we do females, simply because of biology, not… I think that's the only reason that would even matter. … I mean gynecological oncology would be different, or maybe with prostate cancer. I don't see prostate cancer. You know, if you actually don't have a prostate. No, I don't see how it would impact, aside from sometimes when patients share when I ask them kind of about their support system at home, and their home life when you try to figure out family dynamics are, but, I can't think of one time where it truly was like a barrier to something?
	27	Academic	Medium	Sorry to be ignorant. Like my ignorance, I didn't think about it in that sense. So, um, you know, we would just have to counsel the best we could. I mean, I have women that wanna still take estrogen, right… That is not, that is not what we recommend. It would, you know, it can really impact your cancer.
	28	Academic	Large	I actually, um, found this very, um, informative as well, you know, and it, and helps me put some, um, you know, line items in my head so that I, we can, you know, I could document it later and, and hopefully, uh, move forward on some of the, um, you know, on, on, on some of the improvements that I was referring to.
Barriers for transgender patients in the clinic				
Billing challenges affecting patient care	29	Academic	Large	When, when we're, when we're going into the billing and coding modules, um, when using EPIC, the information that's at that encounter level is, um, tied to the patient level information. And what I mean by that is that if it's, if a, if a, if a trans, if a patient that's going through a, a transitional phase, um, and going into, um, that type of change, um, when their, uh, when their legal sex is male, um, you know, some, some procedures, uh, are not allowed … So in order for that to, in order for the billing or their coding or both, uh, to be successfully completed, that, um, that legal sex temporarily has to be changed. So this, there's challenges like that … it really makes it complex for us to do what we have to do.
	30	Academic	Large	Oh, God. Billing challenges [are] awful. We have people… they've been refusing to do imaging because they've been saying that ovarian cancer isn't a valid diagnosis in a man. Um, so we've had insurance deny everything for some of our patients. We spend a lot of time arguing on the phone that we're ordering the wrong study. It's, uh, it's terrible.
Staff errors	31	Academic	Large	There's limited banner space on the EMR, so, uh, when there was a gender discordant patient, we had a hard time noting that. Um, so now there's a pop‐up that shows up. It's not the world's most obvious pop‐up, but it's a pop‐up nonetheless. But the other day, for instance, I was in pre‐op and there was a patient with gender discordancy who, um, the nurse did not pick up on the pop‐up. She just cleared the pop‐up without even reading it and then asked for a beta HCG on the trans woman, um, which was, you know, awkward obviously.
	32	Academic	Large	Cuz some people will come in and they will say, you know, my name isn't [name], I go by [name], but they still very much look like a phenotypic female. And so it can be a little bit challenging, I think, just for us, as on autopilot, you know, in terms of if you have a busy day and you're seeing 50 people, sometimes you don't always think of that sort of stuff. Um, but I mean, anyone can see that anyone who has access to that patient's chart or is opening up that patient's chart can see that information pretty easily.
Lack of disclosure among transgender patients	33	Nonacademic	Small	I've had trans men who just have never mentioned it or felt comfortable bringing it up. But they need to still get screened for mammograms.

### 3.1 Theme 1: Barriers to SOGI Data Collection

The first emergent theme was barriers to SOGI data collection. Subthemes included the role of stigma and bias, generational variations in comfort discussing SOGI, geopolitical variations in comfort discussing SOGI, fragmented data collection processes and limited access to data once documented, competing priorities, and institutional inertia.

#### 3.1.1 Stigma and Bias

Interviewees noted the implicit and explicit bias of health care team members as a significant challenge. While views varied widely depending on location and culture of the oncology care setting, several interviewees indicated that bias and personal beliefs of staff could hinder SOGI data collection (Q1). One respondent stated they thought patients might withhold SOGI data due to perceived stigma from healthcare staff, referencing “social stigma” (Q2).

#### 3.1.2 Generational Variations in Comfort Level Discussing SOGI

Many interviewees noted generational differences in comfort level discussing SOGI, with younger generations welcoming change and older generations of healthcare staff and patients being less comfortable. One respondent at a rural, academic medical center described their center's population as older and indicated that older patients sometimes were confused when asked SOGI questions (Q3). One interviewee at a Midwestern academic hospital indicated older generations of doctors had a restricted view of SOGI in their practice, saying that “older doctors” may “have a harder time talking about those things [SOGI]” (Q4).

Alternatively, interviewees mentioned that younger generations of staff and patients were more open and aware of SOGI differences. An interviewee at an academic center in the West spoke about younger generations' openness regarding SOGI (Q5). Another respondent from a nonacademic, Southern practice mentioned that most of their scribes are Generation Z and identify as part of the LGBTQI community and that openness about SOGI was the norm (Q6).

#### 3.1.3 Geopolitical Variations in Comfort Discussing SOGI

Interviewees noted geopolitical differences based on the majority culture of a particular region. At sites where SOGI data were being implemented, many interviewees reported “really positive feedback from patients” and no negative responses. At these sites, interviewees described a culture where personnel were on board with SOGI data collection, saying that the resistance to data collection was “minimal” (Q7).

Respondents from conservative areas—particularly in the South—described heteronormative institutional cultures and explicit bias among colleagues. These regional influences shaped comfort levels with SOGI data collection and routine clinical behavior, such as misgendering or dismissive remarks about transgender patients (Q8). However, even in a progressive region, a clinician shared that despite expectations of inclusion, their partner was misgendered throughout an interview process in a major West Coast city (Q9).

#### 3.1.4 Polarized Sociopolitical Context

The larger political landscape in the US and its impact on LGBTQI individuals was also highlighted as a concern. Participants described the national political environment as a factor that shaped staff attitudes and institutional willingness to engage in SOGI data collection. Several interviewees cited ongoing legislative efforts and polarizing rhetoric as causing fear, discomfort, or hesitation around addressing LGBTQI identities in clinical settings (Q10–Q11). Despite external pressures, one participant suggested that institutional mandates—such as standardized data fields or required forms—could help overcome resistance, even in politically challenging environments (Q11). Some staff worried that older patients would react negatively to being asked about SOGI, particularly in more conservative geopolitical contexts (Table, Q12–Q14).

#### 3.1.5 Heterogeneous, Fragmented Data Collection and Access

Another key barrier to SOGI data collection was fragmentation and heterogeneity in demographic data collection. One respondent described a lack of a centralized workflow, which included data collection through self‐report, intake website, or form, and front‐desk staff. Several participants suggested that the process for collecting SOGI data felt “disjointed,” noting that the lack of a centralized workflow led to inconsistent or incomplete data entry. Patient self‐report, particularly through electronic systems, was commonly noted as a potential facilitator for accurate and complete data collection (Q15–Q16).

Some respondents highlighted challenges of existing EHRs, especially during transition periods from paper‐based workflows. One participant from an academic center said that unless staff are specifically assigned to input data in structured EHR fields integrated across data record systems, SOGI fields can easily be overlooked (Q17).

#### 3.1.6 Competing Priorities

Among sites that did not prioritize SOGI data collection, concerns about time and competing priorities among healthcare staff were noted. One participant from an academic cancer center mentioned the relative priority of SOGI data collection as being low compared to patient social needs, such as food and housing (Q18).

Likewise, a respondent from an academic cancer center indicated that staff perception of SOGI data was not a barrier to its collection, but resistance arose from a perceived increase in staff workload and pressures to maintain time benchmarks during registration (Q18–19).

#### 3.1.7 Institutional Inertia

When asked about barriers that prevented SOGI data collection from being prioritized, a staff member at an academic cancer center reflected on the challenge of implementing change that would facilitate SOGI data collection, citing “institutional inertia” and “a bunch of attorneys” that would need to approve any changes (Q20). Another respondent described the complex bureaucratic procedures to update forms to have more inclusive language, noting the challenges and multi‐step approval process even for seemingly simple changes (Q21).

### 3.2 Theme 2: Importance of Exposure to Understanding SOGI Relevance

A second emergent theme in our data was the importance of understanding how SOGI was relevant to clinical care.

#### 3.2.1 Varying Perceptions of SOGI Relevance to Clinical Practice

A major difference between sites that did and did not collect SOGI data routinely was varying perceptions on the relevance of SOGI data to clinical practice. A few respondents indicated that SOGI seemed irrelevant to clinical practice (Q22‐Q23) and thus was not collected.

#### 3.2.2 Need for Education on SOGI Data Collection

Several interviewees indicated that staff needed education to understand the importance of SOGI data collection. Education was seen as essential for shifting attitudes and building confidence among clinicians and staff in collecting these data (Q24). Even in well‐resourced, academic settings, there was discomfort in asking about SOGI (Q25). In response, some institutions implemented focused education sessions, including presentations by LGBTQI patients and clinicians, to increase cultural competence and normalize inclusive communication practices (Q25).

#### 3.2.3 Emerging Perception of Relevance of SOGI Through Exposure

Some interviewees who did not initially see the relevance of SOGI data to clinical practice seemed to identify its relevance in the process of being interviewed. For example, a few respondents initially indicated that they perceived SOGI to be irrelevant to the care they provide, but then subsequently described how SOGI data could impact endocrine therapy decisions, prostate and gynecological care, and the assessment of hormone use among transgender patients (Q26–Q27). One respondent specifically noted how important it was to know if cisgender women were taking estrogen, and in the process of explaining this, realized that hormone use would also be very important to know for transgender patients (Q27). Another respondent said the interview prompted them to think about the role of SOGI data in cancer care practice differently and reflect on how SOGI information could be integrated into clinical documentation (Q28).

### 3.3 Theme 3: Barriers for Transgender Patients in the Clinic

A final theme that emerged was specific challenges that transgender patients face in oncology settings, including insurance and billing systems, documentation errors, and fear of disclosing gender identity.

#### 3.3.1 Billing Challenges Affecting Patient Care

Participants described navigating billing systems that rely on sex assigned at birth for both coding procedures and diagnostics. In some cases, clinicians were required to temporarily alter a patient's recorded sex in the EHR for the patient to receive insurance coverage for necessary imaging and treatments. Other participants noted denials for “gendered” procedures (e.g., pelvic ultrasounds, mammograms) in transgender patients, which often result in time‐consuming appeals (Q29–Q30).

#### 3.3.2 Staff Errors

Staff errors were mentioned as a barrier to the use of SOGI data in clinical practice. One respondent described how the number of pop‐ups in their EHR led to staff dismissing the pop‐ups without looking at the information. Frequent alerts led some staff to dismiss critical information (i.e., alert fatigue), sometimes resulting in inappropriate lab orders. For example, a participant at a large, academic cancer center brought up an instance where a beta human chorionic gonadotropin test, typically used to confirm pregnancy, was requested on a transgender woman because of the missed pop‐up (Q31). The fast pace of clinics was a factor in overlooking affirmed names or pronouns, even when the correct information was documented in the patient chart (Q32).

#### 3.3.3 Lack of Disclosure Among Transgender Patients

Some interviewees noted that transgender patients withheld disclosure of gender identity due to perceived and/or anticipated bias or discrimination, leading to missed screening and care. For example, one participant noted instances where transgender men did not disclose their anatomy and thus were not offered mammograms or other appropriate screenings (Q33).

## 4 Discussion

This study represents the largest known qualitative analysis of themes and perspectives on SOGI data collection in oncology practice across the United States. Our findings highlight complex and often conflicting staff member attitudes, data collection processes, and structural barriers that shape how—and whether—SOGI data are collected. These results underscore the need for organizational commitment, workforce training, and established workflows to ensure that LGBTQI patients receive inclusive care.

Stigma and bias were the most cited barriers to SOGI data collection, ranging from implicit discomfort to explicit resistance among staff in oncology clinics. Previous studies have identified similar barriers to data collection, indicating that personal beliefs and cultural norms can negatively impact the collection of SOGI data [15, 16, 17]. Interviewees specifically noted that discomfort and unfamiliarity with how and why to ask patients about SOGI hinder the systematic collection of these data. Notably, younger staff members generally demonstrated greater comfort and willingness to engage in conversations about SOGI as well as in training or mentorship programs to increase overall LGBTQI competency. Thus, identifying younger clinical champions may serve as a facilitator for focused interventions to improve SOGI data collection.

Concerns about patient comfort in being asked about SOGI highlighted the legacy of mistrust between members of the LGBTQI community and the healthcare system [18, 19, 20]. Prior research has illustrated that patients sometimes choose not to disclose SOGI due to “awkward choreography” around disclosure of SOGI in cancer care settings [19], fear of rejection or suboptimal care [20]. There is particular mistrust among transgender and gender nonconforming patients due to negative past experiences and pathologization of gender identity [21]. For example, terms such as “gender dysphoria syndrome,” “transsexualism,” and “transgenderism” consistently show up on problem lists in electronic medical records [21]. Some patients have requested that their identity be “off the record” with staff and medical professionals [21].

Approaches that build trust and secure the confidentiality of patient SOGI information are critical. The Multi‐Regional Clinical Trials Center of Brigham and Women's Hospital and Harvard have recently updated an *LGBTQIA+ Inclusion by Design in Clinical Research Toolkit* [22]. The toolkit provides examples of inclusive imagery and language, a SOGI data collection checklist (including opting out of disclosure for any participant), privacy considerations, and questions that LGBTQIA+ participants may wish to ask a research team [22].

In our study, system inertia was also reported to delay or prevent the adoption of inclusive data practices. Streamlining the process of making changes to forms, workflows, and EHR templates—especially when those changes are aligned with inclusive care practices—may facilitate more widespread and sustainable SOGI data collection. Adjustments to time expectations for patient intake and efficient ways of collecting data may also be needed to facilitate SOGI data collection. Ironically, while some oncology staff did not perceive SOGI as clinically relevant, they identified scenarios in which knowledge of patient SOGI was fundamental to delivering effective clinical care. Staff with less exposure to patients who identified as LGBTQI sometimes expressed concerns about social determinants of health, financial concerns, and logistical barriers being more pressing than identifying SOGI data for patients.

The last decade has seen increasing SOGI data collection in response to national calls for better data to inform clinical practice [4, 5, 6, 7, 8, 9, 10, 11, 12, 13, 14, 15, 16]. Data collection for the present study was conducted in 2022–2023. In 2025, the LGBTQI community is facing stronger policy headwinds, including an ever‐growing number of anti‐LGBTQI state‐ and federal‐level policies. The year 2025 saw the highest number of U.S. state‐level anti‐LGBTQI bills ever [23, 24]. Numerous federal executive orders [25] and restrictive federal policies endanger healthcare access for transgender patients and instill fear among institutional leadership, potentially deterring data collection [26]. While the full implications remain uncertain, these policy changes underscore the urgent need to advocate for inclusive, safe, confidential, and equitable cancer care. Transgender patients need comprehensive healthcare both related to and unrelated to their gender identity. Clinicians and staff within oncology practices play a critical role in ensuring respectful and affirming care in an increasingly hostile sociopolitical environment.

## 5 Implications

To address the barriers identified, institutional policies should prioritize: (1) education regarding the relevance and importance of SOGI to clinical care management, and (2) safe, confidential SOGI data collection processes that are safeguarded from disclosure to politically‐motivated elected officials. Institutions should provide clear guidance for implementation and ensure that staff have the necessary time, training, and resources to incorporate these practices into their workflows. Furthermore, clear planning to ensure patients understand the risks of disclosing SOGI, explicit nondisclosure options, and procedures to maintain the confidentiality of SOGI data are imperative, particularly at this time [22].

In addition, while SOGI data collection has expanded in the last decade, participant comments regarding alert fatigue suggest that streamlined approaches that prioritize what is most urgent and contextualize SOGI when clinically relevant will be important considerations for future clinical support systems design [27].

## 6 Limitations

We aimed to interview three individuals at each institution, knowing that different roles would have different insights into SOGI data collection. However, in some instances, we were only able to interview one or two individuals at an institution. In one case, we had four perspectives represented in one setting. Additionally, as with all cross‐sectional research, our data reflect a prior snapshot in time and may not fully reflect challenges in the current context. Caution should be exercised when attempting to apply findings from these cases to other settings, as these results do not necessarily generalize to other contexts. With the rapidly evolving policy landscape, changes to cultural norms may lead to greater challenges in SOGI data collection in the near future. As legislative and cultural landscapes continue to shift, oncology practices have the opportunity to lead ethical health care by acting decisively to ensure LGBTQI patients receive respectful and informed care. Lastly, five of the authors are members of the LGBTQI community, and we realize that this may influence the interpretation of these results.

## 7 Conclusions

The findings of this large, national qualitative study highlight diverse challenges associated with SOGI data collection across cancer care settings. Structural limitations as well as personal bias and interpersonal dynamics were barriers to SOGI data collection and high‐quality, gender‐affirming oncology care for transgender patients. Prior research on this dataset describes facilitators for SOGI data implementation [14]. Future studies should evaluate the effectiveness of implementation strategies, building on the factors identified in this analysis to optimize SOGI data collection in oncology settings.

## Author Contributions

Conceptualization: M.L.P.‐C. Data curation: M.L.P.‐C., D.R., B.G., M.E.I. Formal analysis: M.L.P.‐C., D.R., B.G., M.E.I. Funding acquisition: M.L.P.‐C., C.K., M.A.M. Investigation: M.L.P.‐C., C.K., M.A.M. Methodology: M.L.P.‐C. Project administration: M.L.P.‐C. Supervision: M.L.P.‐C. Writing original draft: M.L.P.‐C., D.R., B.G., M.E.I. Writing reviewing and editing: M.L.P.‐C., M.A.M., C.K., E.J.M.

## Ethics Statement

This study was approved by the Institutional Review Boards of the George Washington University (IRB #NCR224177), UT Southwestern Medical Center (IRB #STU‐2022‐1122), and the University of Rochester Medical Center (IRB #STUDY00007236). All participants provided verbal informed consent prior to participation.

## Consent

Verbal consent was obtained from all participants prior to the interviews being conducted.

## Conflicts of Interest

The authors declare no conflicts of interest.

## Supporting information


**Data S1:** Supporting Table. Census Region and Associated State: 50 States and Washington D.C. Are Classified Into four Regions Across the United States.

## Data Availability

The data that support the findings of this study are available from the corresponding author upon reasonable request.
