# Supplementary material for: Sexual Orientation and Gender Identity Data in Oncology: Thematic Analysis From a National Qualitative Interview Study
Source: Cancer Med. 2025 Nov 20;14(22):e71360. doi: 10.1002/cam4.71360 (PMC12631534; doi:10.1002/cam4.71360)
Supplement: Supplementary file 1 — Data S1: Supporting Table. Census Region and Associated State: 50 States and Washington D.C. Are Classified Into four Regions Across the United States. [file CAM4-14-e71360-s001.docx]

**Supplement Table**. Census Region and Associated States

| **Census Region** | | | |
| --- | --- | --- | --- |
| Northeast | Midwest | South | West |
| Connecticut | Illinois | Alabama | Alaska |
| Maine | Indiana | Arkansas | Arizona |
| Massachusetts | Iowa | Delaware | California |
| New Hampshire | Kansas | Distict of Columbia | Colorado |
| New Jersey | Michigan | Florida | Hawaii |
| New York | Minnesota | Georgia | Idaho |
| Pennsylvania | Missouri | Kentucky | Montana |
| Rhode Island | Nebraska | Louisiana | Nevada |
| Vermont | North Dakota | Maryland | New Mexico |
|  | Ohio | Mississippi | Oregon |
|  | South Dakota | North Carolina | Utah |
|  | Wisconsin | Oklahoma | Washington |
|  |  | South Carolina | Wyoming |
|  |  | Tennessee  Texas  Virginia  West Virginia |  |
